# Melioidosis in Birds and *Burkholderia pseudomallei* Dispersal, Australia

**DOI:** 10.3201/eid1707.100707

**Published:** 2011-07

**Authors:** Vanya Hampton, Mirjam Kaestli, Mark Mayo, Jodie Low Choy, Glenda Harrington, Leisha Richardson, Suresh Benedict, Richard Noske, Stephen T. Garnett, Daniel Godoy, Brian G. Spratt, Bart J. Currie

**Affiliations:** Author affiliations: Menzies School of Health Research–Charles Darwin University, Darwin, Northern Territory, Australia (V. Hampton, M. Kaestli, M. Mayo, J. Low Choy, G. Harrington, L. Richardson, B.J. Currie);; School for Environmental Research–Charles Darwin University, Darwin (R. Noske, S.T. Garnett);; Berrimah Veterinary Laboratories, Darwin (S. Benedict);; Imperial College, London, UK (D. Godoy, B.G. Spratt)

**Keywords:** meliodosis, Burkholderia pseudomallei, Australia, birds, zoonoses, dispersal, letter

**To the Editor:** Melioidosis is an emerging infectious disease of humans and animals caused by the gram-negative bacterium *Burkholderia pseudomallei*, which inhabits soil and surface water in the disease-endemic regions of Southeast Asia and northern Australia (*1*). The aim of this study was to assess the potential for birds to spread *B. pseudomallei*. Birds are known carriers of various human pathogens, including influenza viruses, West Nile virus, *Campylobacter jejuni*, and antimicrobial drug–resistant *Escherichia coli* (*2*).

During February–August 2007, we conducted a survey to determine *B. pseudomallei* carriage in 110 wild native finches and doves from the melioidosis-endemic Darwin region, Northern Territory, Australia. Swab specimens from the beaks, feet, cloacae, and feces were cultured for *B. pseudomallei* as described (*3*). One healthy (normal physical appearance, weight, and hematocrit) native peaceful dove (*Geopelia placida*) at a coastal nature reserve was found to carry *B. pseudomallei* in its beak. The peaceful dove is a common, sedentary, ground-foraging species in the Darwin region. *B. pseudomallei* was not detected in environmental samples from the capture site, but *B. pseudomallei* is known to occur within 3 km of the capture site (*4*), the typical movement range for this bird species. On multilocus sequence typing (MLST) (*5*), the *B. pseudomallei* isolate was identified as sequence type (ST) 144, which we have previously found in humans and soil within 30 km of the site.

Numerous cases of melioidosis in birds have been documented (Technical Appendix). However, these are mostly birds in captivity and often exotic to the location, suggesting potential reduced immunity. Little is known about melioidosis in wild birds. In Sabah, Malaysia, only 1 of 440 wild birds admitted to a research center over 9 years was found to have melioidosis (*6*).

Although birds are endotherms, with high metabolic rates and body temperature (40°C–43°C) protecting them from many diseases, some birds appear more susceptible to melioidosis. Indeed, high body temperature would not deter *B. pseudomallei*, which is routinely cultured at 42°C and at this temperature shows increased expression of a signal transduction system, which is involved in pathogenesis (*7*).

Examples of birds with fatal melioidosis in our studies in the Darwin region include a domesticated emu in 2009 with *B. pseudomallei* cultured from brain tissue and a chicken in 2007 with *B. pseudomallei* cultured from facial abscesses. In 2007, an outbreak of melioidosis occurred in an aviary; 4 imported exotic yellow-bibbed lorikeets (*Lorius chlorocercus)* died within months of arriving from a breeder in South Australia. On necropsy, the birds showed nodules throughout the liver and spleen (Figure). *B. pseudomallei* was cultured from the liver, spleen, crop, beak, and rectum. At the aviary, *B. pseudomallei* was also found in water from sprinklers, the water bore head, soil next to the bore, and the drain of the aviary. The unchlorinated sprinkler system used to cool the aviary was identified as the likely source of infection. MLST and 4-locus multilocus variable-number tandem repeat analysis (*8*) suggested a point-source outbreak with an identical 4-locus multilocus variable-number tandem repeat analysis pattern and ST for all *B. pseudomallei* isolated from the diseased birds and the sprinkler system. The ST was novel (ST673), with no single-locus variants in the global MLST dataset.

**Figure:**
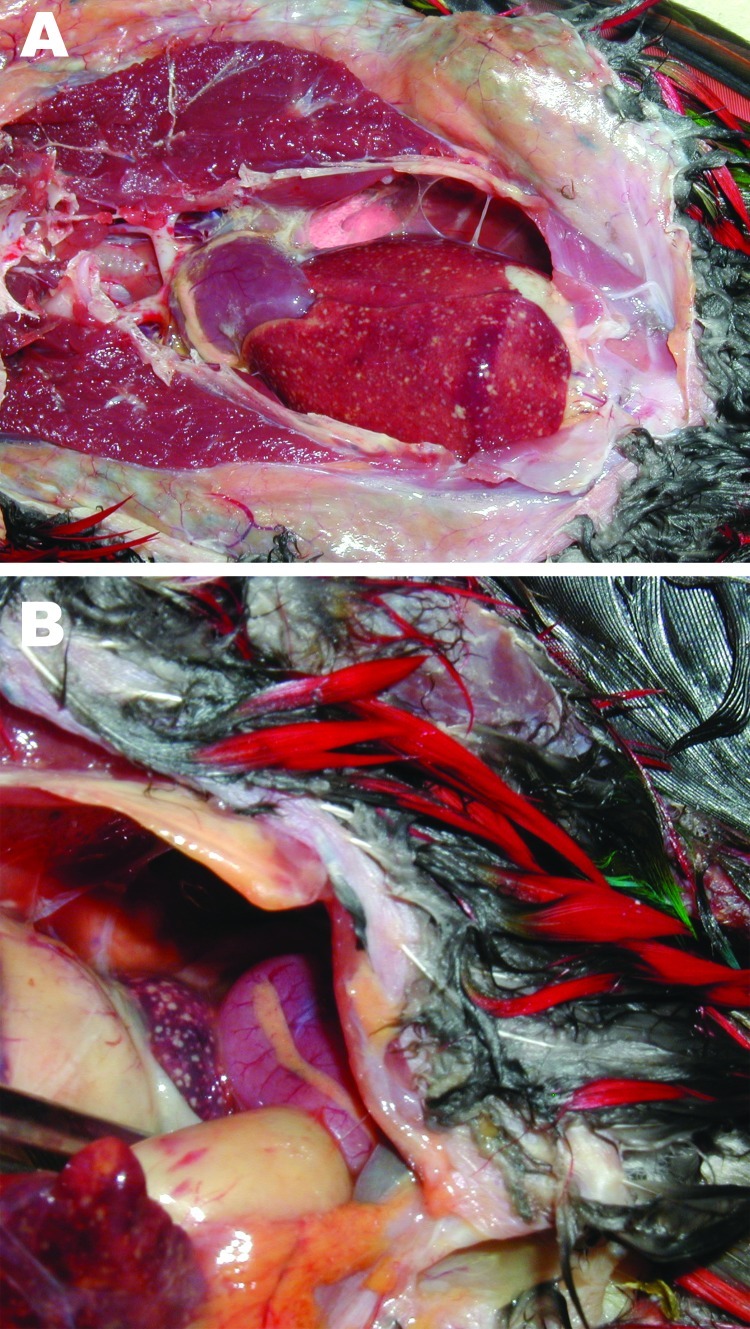
Images from necropsy of yellow-bibbed lorikeet that died of melioidosis, showing multiple diffuse nodular lesions in the liver (A) and spleen (B). Photographs by Jodie Low Choy.

Although an infected exotic or captive bird is likely to quickly die from melioidosis, our survey suggests that native birds are not very susceptible to infection with *B. pseudomallei* and resulting disease*.* Further studies are required to quantify the carriage of *B. pseudomallei* in wild native birds in melioidosis-endemic locations. Nevertheless, although no direct proof exists for spread of *B. pseudomallei* by birds, our finding of an asymptomatic native bird with *B. pseudomallei* in its beak supports the hypothesis of potential dispersal of these bacteria by birds from melioidosis-endemic regions to previously uncontaminated areas. For instance, carriage by birds could explain the introduction of *B. pseudomallei* to New Caledonia in the Pacific, 2,000 km east of Australia. *B. pseudomallei* strains from New Caledonia are related by MLST to Australian strains; 1 strain is a single-locus variant of a strain from Australia’s east coast (*9*). Vagrant water birds are known to irregularly disperse from eastern tropical Australia to the southwestern Pacific, presumably driven by drought and offshore winds (G. Dutson, pers. comm.). Thus, *B. pseudomallei* could have been introduced to New Caledonia by an infected bird that flew there from northeastern Australia.

In summary, melioidosis is uncommon in wild birds but occurs in captive or exotic birds brought to melioidosis-endemic locations. Asymptomatic carriage of *B. pseudomallei* can occur in wild birds but appears to be unusual. We believe the risk for spread of *B. pseudomallei* by birds is low, but such occurrence does provide a possible explanation for the spread of melioidosis from Australia to offshore islands.

## Supplementary Material

Technical Appendix Birds with reported melioidosis or carriage of Burkholderia pseudomallei in previous publications and this report.
