# Supplementary material for: Melioidosis in Birds and Burkholderia pseudomallei Dispersal, Australia
Source: Emerg Infect Dis. 2011 Jul;17(7):1310–2. doi: 10.3201/eid1707.100707 (PMC3381411; doi:10.3201/eid1707.100707)
Supplement: Technical Appendix — Birds with reported melioidosis or carriage of Burkholderia pseudomallei in previous publications and this report. [file 10-0707-Techapp.pdf]

Article DOI: 10.3201/eid1707.100707

Melioidosis in Birds and *Burkholderia pseudomallei* Dispersal, Australia

Technical Appendix

Birds with reported melioidosis or carriage of *Burkholderia pseudomallei* in previous publications and this report

Common name	Species name	Origin of bird species	Residence of diseased bird	Captive status	*B. pseudomallei* culture site	Reference
Parrot	NA	NA	Malaysia	NA	NA	(*1*)
Sulfur-crested cockatoo	*Cacactua galerita*	Australia	Townsville, Australia	Captive	Necropsy	(*2*)
Galah	*Eolophus roseicapillus*	Australia	Townsville, Australia	Captive	Necropsy	(*3*)
Unknown native birds		Australia	Queensland, Australia	Captive	Necropsy	(*4*)
Macaroni penguin	*Eudyptes chrysolophus*	Maquarie Island	Hong Kong	Captive	Necropsy	(*5*)
Cassowary	*Casuarius* sp	Australia	Singapore	Captive	NA	(*6*)
Palm cockatoo	*Probosciger aterrimus*	Australia, New Guinea	Singapore	Captive	NA	(*6*)
Crown pigeon	Crowned pigeon: *Goura* sp	NA, New Guinea	Singapore	Captive	NA	(*6*)
Chicken	*Gallus gallus*	Southeast Asia	France	Captive	Experimental injection/necropsy	(7)
Scarlet macaw	*Ara macao*	Central America	Hong Kong	Captive	NA	(*8*)
Zebra dove	*Geopelia striata*	Southeast Asia	Hong Kong	Captive	NA	(*8*)
Unknown bird	NA	NA	Malaysia	Wild	NA	(*9*)
African gray parrot	*Psittacus erithacus*	Africa	Darwin, Australia	Captive	Necropsy	This report
Yellow-bibbed lorikeet	*Lorius chlorocercus*	Solomon Islands	Darwin, Australia	Captive	Necropsy	This report
Peaceful dove†	*Geopelia placida*	Australia	Darwin, Australia	Wild	Beak swab	This report
Chicken	*Gallus gallus*	Southeast Asia	Darwin, Australia	Captive	Skin lesions	This report
Emu	*Dromaius novaehollandiae*	Australia	Darwin, Australia	Captive	Brain	This report

*NA, information not available.
†Asymptomatic bird.

References

1. Lim SY, Tan BE. *Actinobacillus whitmori* isolated from a parrot. Kajian Veterinaire. 1967;44–8.

2. Thomas AD, Wilson AJ, Aubrey JN. Melioidosis in sulphur-crested cockatoo (*Cacatua galenta*). Aust Vet J. 1978;54:306–7. PubMed doi:10.1111/j.1751-0813.1978.tb02466.x

3. Thomas AD, Norton J, Pott B. Melioidosis in a galah (*Cacatua roseicapilla*). Aust Vet J. 1980;56:192–3. PubMed doi:10.1111/j.1751-0813.1980.tb05680.x

4. Thomas AD. Prevalence of melioidosis in animals in northern Queensland. Aust Vet J. 1981;57:146–8. PubMed doi:10.1111/j.1751-0813.1981.tb00494.x

5. MacKnight K, Chow D, See B, Vedros N. Melioidosis in a macaroni penguin *Eudyptes chysolophus.* Dis Aquat Organ. 1990;9:105–7. doi:10.3354/dao009105

6. Yap EH, Thong TW, Tan AL, Yeo M, Tan HC, Loh H, et al. Comparison of *Pseudomonas pseudomallei* from humans, animals, soil and water by restriction endonuclease analysis. Singapore Med J. 1995;36:60–2. PubMed

7. Vesselinova A, Najdenski H, Nikolova S, Kussovski V. Experimental melioidosis in hens. Zentralbl Veterinarmed B. 1996;43:371–8. PubMed

8. Godoy D, Randle G, Simpson A, Aanensen D, Pitt T, Kinoshita R, et al. Multilocus sequence typing and evolutionary relationships among the causative agents of melioidosis and glanders, *Burkholderia pseudomallei* and *Burkholderia mallei.* J Clin Microbiol. 2003;41:2068–79. PubMed doi:10.1128/JCM.41.5.2068-2079.2003

9. Ouadah A, Zahedi M, Perumal R. Animal melioidosis surveillance in Sabah. The Internet Journal of Veterinary Medicine. 2007;2. http://www.ispub.com/journal/the_internet_journal_of_veterinary_medicine/volume_2_number_2_2/article/animal_melioidosis_surveillance_in_sabah.html
